# Unique quantitative Symbiodiniaceae signature of coral colonies revealed through spatio-temporal survey in Moorea

**DOI:** 10.1038/s41598-019-44017-5

**Published:** 2019-05-28

**Authors:** Héloïse Rouzé, Gaël Lecellier, Xavier Pochon, Gergely Torda, Véronique Berteaux-Lecellier

**Affiliations:** 1 USR3278 CRIOBE, Labex CORAIL, BP 1013 Papetoai, 98729 Moorea, French Polynesia; 2 UMR250/9220 ENTROPIE, Labex CORAIL, 101, promenade Roger-Laroque, BP A5 98848, Noumea, cedex New Caledonia; 3 Université de Paris-Saclay, UVSQ, 55 Avenue de Paris, 78035 Versailles, Cedex France; 4 Coastal and Freshwater Group, Cawthron Institute, 98 Halifax Street East, 7010 Nelson, New Zealand; 5 Institute of Marine Science, University of Auckland, Warkworth, 0941 New Zealand; 6 Australian Research Council Centre of Excellence for Coral Reef Studies, James Cook University, Townsville, Australia

**Keywords:** Molecular biology, Ecology

## Abstract

One of the mechanisms of rapid adaptation or acclimatization to environmental changes in corals is through the dynamics of the composition of their associated endosymbiotic Symbiodiniaceae community. The various species of these dinoflagellates are characterized by different biological properties, some of which can confer stress tolerance to the coral host. Compelling evidence indicates that the corals’ Symbiodiniaceae community can change via shuffling and/or switching but the ecological relevance and the governance of these processes remain elusive. Using a qPCR approach to follow the dynamics of Symbiodiniaceae genera in tagged colonies of three coral species over a 10–18 month period, we detected putative genus-level switching of algal symbionts, with coral species-specific rates of occurrence. However, the dynamics of the corals’ Symbiodiniaceae community composition was not driven by environmental parameters. On the contrary, putative shuffling event were observed in two coral species during anomalous seawater temperatures and nutrient concentrations. Most notably, our results reveal that a suit of permanent Symbiodiniaceae genera is maintained in each colony in a specific range of quantities, giving a unique ‘Symbiodiniaceae signature’ to the host. This individual signature, together with sporadic symbiont switching may account for the intra-specific differences in resistance and resilience observed during environmental anomalies.

## Introduction

Dinoflagellate algae from the family Symbiodiniaceae are one of the keystone taxa for coral reef ecosystems. Their importance lies in that in tandem to living free in the environment^1,2^, they also form photo-symbiotic associations with corals and several other invertebrates (e.g.^3,4^). Their extraordinary diversity encompasses at least nine major lineages^5^, labelled A to I in the literature some of which were recently erected to genus level^5^: *Symbiodinium* (clade A), *Breviolum* (clade B), *Cladocopium* (clade C) and *Durusdinium* (clade D). Moreover, each lineage encompasses multiple distinct genetic types^6,7^. The acquisition of Symbiodiniaceae by corals is initiated during early life stages via vertical/maternal transfer or through horizontal pathways (reviewed in^8^). This fine-tuned partnership between the metazoan coral and the dinoflagellates enables the coral-guild (i.e. the holobiont) to thrive in oligotrophic waters (e.g.^9–14^). The collapse of this symbiosis is becoming a common global phenomenon as environmental anomalies are becoming more and more frequent and severe, leading to mass coral bleaching and mortality events globally^15–18^.

The coral holobiont’s resilience to environmental stressors is in large part based on various genotypes and biological traits^19^. This includes the complex interaction and differential physiological responses of symbiotic Symbiodiniaceae populations that may be composed of one single genus or multiple genera and species^20,21^ and the host^22–25^. A plethora of *in situ* and *ex situ* studies have reported highly dynamic coral-Symbiodiniaceae associations in response to environmental changes, contrasts or extremes^26–31^. This is a particularly complex mechanism to study, as the free-living Symbiodiniaceae communities may be altered depending on habitat quality^32,33^. Shifts that occur over the coral’s ontogeny have been related to physiological states (e.g. diseased vs. healthy, temperature resilience)^34–37^. These ecological observations support the idea that flexibility in the coral-Symbiodiniaceae partnership enables the coral holobiont to adapt rapidly to environmental stressors^38^, a paradigm that is encapsulated in the Adaptive Bleaching Hypothesis^39^. In contrast, the analysis of ancient DNA from octocoral species revealed a stable coral host-Symbiodiniaceae association over the last century^40^. Moreover, comparative genomic and transcriptomic analyses showed evidence of a metabolic continuum between the genomes of corals and associated symbionts, supporting the hypothesis that co-evolutionary mechanisms in corals play important roles in the maintenance and adaptation of the symbiosis^41–43^. In line with these observations, coral-Symbiodiniaceae associations can exhibit a high degree of specificity^44–47^ at the level of both genera and species, and there is a lack of evidence for adult coral colonies to form stable partnerships with newly acquired exogenous Symbiodiniaceae^48,49^. Therefore, corals have to balance two apparently incompatible traits: Symbiodiniaceae genus fidelity versus Symbiodiniaceae genus flexibility^50^.

Deciphering the extent of symbiont change potentially occurring among different coral species through shuffling (intrinsic changes) and/or switching (extrinsic changes) of Symbiodiniaceae communities^39^, requires the consideration of cryptic taxonomic units present at trace levels^21,51–53^. While an increasing attention has been directed towards these symbiotic cryptic populations since the development of high-sensitivity molecular techniques such as real-time quantitative Polymerase Chain Reaction (qPCR;^21,51^) and Next-Generation Sequencing^54–59^, their ecological role in the holobiont’s performance is still poorly understood. To date, studies investigating cryptic Symbiodiniaceae populations have mostly focused on the presumably stress tolerant genus *Durusdinium*, and the potential for change in the relative abundance of pre-existing Symbiodiniaceae genotypes in response to heat stress^23,28,31,53,56,60,61^. Recent studies also using random sampling surveys revealed that possible *de novo* acquisition of exogenous Symbiodiniaceae species can occur associated with environmental changes^47,56^. In contrast, a long-term survey of diseased *A. cytherea* corals found that the acquisition of unusual Symbiodiniaceae genera by corals facing environmental stressors is sporadic rather than specific^36^.

In the present study we evaluate the potential of Symbiodiniaceae genus dynamics as a rapid adaptive mechanism for the coral holobiont. We study the composition of Symbiodiniaceae communities and their quantitative regulation in tagged colonies of three coral species over a period of 10–18 months, among environmentally contrasting locations of Moorea (French Polynesia).

## Material and Methods

### Monitoring design: reefs and corals

**Figure 1:**
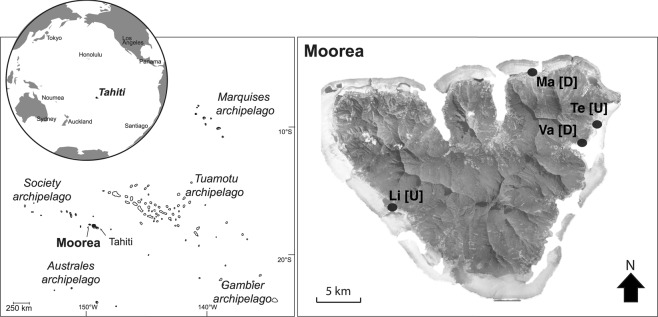
Map of Moorea island (Archipelago of society, French Polynesia) and the locations of the fringing reefs studied: disturbed [D] sites Vaiare (Va) and Maharepa (Ma) versus undisturbed [U] sites Linareva (Li) and Teavaro (Te) modified from^50^.

Sampling was conducted in the Moorea lagoon, in the Society Archipelago, French Polynesia (Fig. 1) from February 2011 to August 2012, i.e. our study period included two wet (November to April) and two dry (May to October) seasons (Table S2). The spatio-temporal dynamics of Symbiodiniaceae genera were investigated on three dominant Pacific coral species (*Pocillopora acuta*, *Acropora cytherea*, and *Porites rus*) with contrasting biological traits (Table 1). Sample collection took place every two months in four inshore fringing reef locations (1–2 m depth; Fig. 1) over a period of 10 months for *P. rus*, 14 months for *A. cytherea*, and 18 months for *P. acuta* (*P. damicornis* type β *sensu*^62^ genetically identified in^50^). At every timepoint, small fragments (0.5–1 cm^3^) were collected from each of 5 to 6 tagged adult colonies per coral species per study site (Table 1). For *Acropora cytherea* 11 colonies correspond to those used by Rouzé *et al*.^36^ and 5 new colonies were added for this study. The four study sites (Fig. 1) were selected for their contrasting environmental conditions: exposed to human activities (Disturbed: D) at Vaiare (in the direct vicinity of a highly frequented ferry wharf) and Maharepa (anthropogenic impacts from hotel facilities, aquatic activities and close to a river mouth subject to intense terrestrial runoffs) versus limited to human activities (Undisturbed: U) at Linareva and Teavaro^63^.

**Table 1:** Biological traits and conditions of the surveys of the tagged coral colonies *Acropora cytherea* (ACY), *Pocillopora acuta* (PAC) and *Porites rus* (PRU).

Species	Biological Traits			Survey	
	Morphology	Resistance	Symb. acquisition	Period	Site (N)
PAC	Branching	Moderate	Vertical	Feb11-Aug12	Li (5), Te (5), Va (5), Ma (5)
PRU	Massive-branching	High	Vertical	Oct11-Aug12	Li (5), Te (5), Va (5), Ma (5)
ACY	Tabular	Low	Horizontal	Jun11-Aug12	Va (5), Li (5)*, LI2 (6)*

Reef sites are coded as follows: Linareva (Li), Teavaro (Te), Vaiare (Va) and Maharepa (Ma) with the number of colonies tagged in each of them (N). Asterisk indicates samples used in Rouzé *et al*. 2016^36^.

Biological and physical environmental parameters (i.e. seawater temperature, nutrients, phytoplankton and sedimentation) were also collected at each timepoint at each location as described in Rouzé *et al*.^63^ (Fig. S1 and Table S2).

### DNA extraction and Symbiodiniaceae/host ratio quantification

The fragments collected from each coral colony were stored individually in 80% ethanol (EtOH) at −20 °C until genomic DNA extraction. All CTAB-based DNA extractions, qPCR assays (i.e. tests for specificity and efficiency of each genus-specific primer) and Symbiodiniaceae/host ratio measurements, including the conditions of qPCR assays, were conducted as described in^50^. Briefly, Symbiodiniaceae from the genus *Symbiodinium*, *Breviolum*, *Cladocopium* and *Durusdinium* and clades E and F^64^ were targeted and quantified using the nuclear ribosomal large subunit (28S rRNA) gene as a template. More specifically, the sensitivity degree of this qPCR method was estimated at the order of 1 algal cell for *Symbiodinium*, *Cladocopium* and *Durusdinium* from assays performed on series of counted symbiotic cells isolated from corals (see^50^), which allows for the detection of background populations. Each sample was analyzed twice on the same plate, as one technical replicate, and averaged when the variation between both Ct values was not exceeding 1. An inter-plate calibrator (i.e. positive control with known concentrations and Ct values: mixture of DNA from different Symbiodiniaceae genera^50^, tested in triplicates (one technical replicate), was added to each plate to calibrate Ct values (performed manually on the MxPro software to set the fluorescent threshold to a fixed Ct value) among different plates of coral DNA samples. The proportion of Symbiodiniacea genera was then estimated by measuring the symbiont/host ratio (S/H ratio) between gene copy numbers of the Symbiodiniaceae 28S and the coral 18S rRNA gene copy numbers in each colony (transformed by log +1). This enabled the simultaneous comparison of Symbiodiniaceae genera within and amongst coral DNA extracts.

### Defining the stability of Symbiodiniaceae communities

Based on the presence over time of each Symbiodiniaceae genus in each surveyed coral colony, we defined an arbitrary ‘genus stability status’, as follows: 1) ‘Permanent’ (detected in more than 80% of sampling timepoints) and 2) ‘non-permanent’ (detected in less than or equal to 80% of sampling timepoints) subdivided into ‘temporal’ (detected in more than two consecutive sampling timepoints) or ‘sporadic’ (detected in less than three consecutive sampling timepoints) based on the global genera distribution compared to random distribution (SI 2).

### Statistical analysis

For each Symbiodiniaceae genus that was quantified in any given sample, the S/H ratios were estimated and log +1 transformed for further analyses. Differences in S/H ratio of stable Symbiodiniaceae genera were tested among coral species and study sites using either analyses of variance (ANOVA) in case of homogeneity of variance; or non-parametric Kruskal-Wallis tests on ranks.

Symbiodiniaceae genus dynamics were estimated for each coral host by the calculation of the difference of S/H ratio of two successive sampling times t_n_ and t_n+1_ (genus i, sampling time n: ∆_genus i,n_ = [S_i_/H]_tn+1_ − [S_i_/H]_tn_). Similarly, the delta values of each environmental parameter (from Table 1 in^63^) were calculated for each period (parameter j: ∆_j_ = [j]t_n+1_ − [j]t_n_), and then combined to consider the overall Environmental Context (EC) at each period. A hierarchical clustering of ECs was done using Euclidian metrics and the Ward method, yielding seven clusters, each characterized by a unique set of environmental variations (Fig. 3).

All statistical analyses were performed using R software (R Foundation for Statistical Computing, version 2.15) using the package ‘*stats*’ for the ANOVAs (normality and homoscedasticity of variances screened) and corresponding pairwise *posthoc* Tukey’s tests or chi-squared tests, and simple or multiple linear regressions. For all analyses, the confidence interval was set to 0.95.

## Results

**Figure 2:**
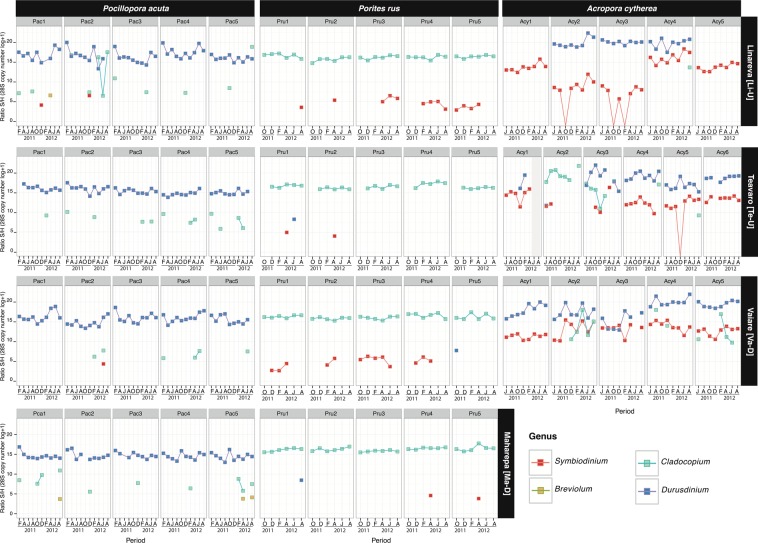
Spatio-temporal survey of Symbiodiniaceae of genus *Symbiodinium*, *Breviolum*, *Cladocopium* and *Durusdinium* associated in 56 tagged coral colonies of 3 coral species (*Pocillopora acuta* [Pac], *Porites rus* [Pru] and *Acropora cytherea* [Acy]) at four reef sites (Linareva [Li], Teavaro [Te], Vaiare [Va] and Maharepa [Ma]) from February 2011 to August 2012. Between 5 and 6 tagged colonies were sampled for each coral species from each study reef site, each represented by a panel (e.g. Pac1 at Li is colony 1 of *P. acuta* at Linareva).

In total, the composition of Symbiodiniaceae genera from 20 *P. acuta*, 20 *P. rus*, and 16 *A. cytherea* colonies were screened over a 10 to 18-month survey at four reef locations (Table 1). Symbiodiniaceae genera *Symbiodinium*, *Cladocopium* and *Durusdinium* were recorded at least once in every coral species, while clades E and F were never recorded. Genus *Breviolum* was exclusively detected in *P. acuta*, and only at three occasions, always as background population representing less than 5% of the relative proportion of total Symbiodiniaceae^47,50,51^ (S/H ratio: <5 28S copy number in log +1, Fig. 2).

### Individual coral colonies exhibit a stable S/H ratio of permanent Symbiodiniaceae genera over time

Symbiodiniaceae genera *Cladocopium* and *Durusdinium* are the sole permanent genera in *P. rus* (*Cladocopium* permanence, hereafter *Cladocopium*-pattern) and *P. acuta* (*Durusdinium* permanence, hereafter *Durusdinium*-pattern), respectively (Fig. 2). *Porites rus* also harbored non-permanent background populations of *Symbiodinium* and *Durusdinium*, while *P. acuta* harbored populations of *Symbiodinium*, *Breviolum* and *Cladocopium*.

In addition to the specificity of Symbiodiniaceae genus-patterns, each host colony was quantified with a specific sustainable S/H ratio range of its associated permanent genera over time (Fig. 2). *Pocillopora acuta* exclusively associated with *Durusdinium*-pattern communities with significant variation among colonies (Table 2) and sites (ANOVA: df = 3, F = 38.391, *P* < 0.001), leading to a total of nine distinct groups (Table 2). The recorded range of S/H ratio of *Durusdinium* were highest in Linareva (mean: 16.6 ± 1.5S/H ratio), lowest in Maharepa (mean: 14.6 ± 0.8S/H ratio) and intermediate at Vaiare and Teavaro (mean: 15.7 ± 1.3 and 15.3 ± 0.8S/H ratio, respectively). In *P. rus* intra-specific variation of *Cladocopium*-pattern community composition was also significant, albeit in lower magnitude (Table 2), with a total of 19 colonies partitioned into five groups varying between 17.2 ± 0.6 to 15.7 ± 0.6S/H ratio without significant site effect (ANOVA: df = 3, F = 2.56, *P* = 0.06). In addition, for the single *P. rus* colony that also harbored *Symbiodinium* as permanent member of its dinoflagellate community (*Cladocopium* and *Symbiodinium*-pattern), the S/H ratio of *Cladocopium* fell within the same range as for four other colonies with *Cladocopium*-pattern (Table 2; Sign.4 at Maharepa, Teavaro and Vaiare). Conversely, intraspecific variation in Symbiodiniaceae community composition was most pronounced in *A. cytherea* (Fig. 2): the majority of colonies (n = 10) simultaneously hosted *Durusdinium* and *Symbiodinium* (*Durusdinium* and *Symbiodinium*-pattern); three colonies hosted only genus *Symbiodinium* (*Symbiodinium*-pattern); two colonies hosted only genus *Durusdinium*, and one colony hosted only genus *Cladocopium*. Among the various symbiotic patterns observed in *A. cytherea*, significantly distinct groups were identified based on the S/H ratios (ANOVA; *Symbiodinium*: df = 12, F = 12.82 and clade D: df = 11, F = 9.3, *p* < 0.001). Table 2 shows 9 distinct groups among the 10 *Symbiodinium* and *Durusdinium*-pattern colonies, 2 distinct groups among the 3 *Symbiodinium*-pattern colonies and 2 distinct groups between 2 *Durusdinium*-pattern colonies. The S/H ratio range of permanent genera was similar for *Symbiodinium* regardless of sites (df = 2, F = 2.004, *P* = 0.141), and even following its punctual loss (e.g. Acy2 at Li  October 2011). In contrast, the S/H ratio range for *Durusdinium* was significantly different among study sites (ANOVA: clade D: df = 2, F = 14.775, *P* < 0.001). Overall, the colonies from Linareva characterized by a genus pattern involving *Durusdinium* displayed the highest ranges of S/H ratio (Table 2; mean ‘genus 1 v’: 19.9 ± 1.0 S/H ratio), when compared to those detected at sites Teavaro and Vaiare (from 15.2 ± 2.1 to 20.1 ± 1.0 S/H ratio; Table 2). *Acropora cytherea* colonies associated with a stable *Cladocopium*-pattern community had a mean S/H ratio of 19.6 ± 1.5.

**Table 2:** Symbiodiniaceae signatures (GROUP) of each surveyed coral colony (in rows) from *Pocillopora acuta, Porites rus*, and *Acropora cytherea*, based on significant differences in the quantities of their permanent Symbiodiniaceae genera (ANOVA analyses on genus quantity among coral hosts and their corresponding *post-hoc* Tukey tests p < 0.05). The significant groups discriminated with the *Post-hoc* Tukey tests are indicated with the following lowercase letters: v, w, x, y, z, i.

Coral-algae	Group	*Post-hoc* TUKEY													Site (ID colony)
		Genus 1					med ± SD	Genus 2						med ± SD	
*Pocillopora acuta*		v	w	x	y	z	*	v	w	x	y	z	i	*	
*Durusdinium*	Sign. 1	•					17.6 ± 1.4								Li (#4)
	Sign. 2	•	•				17.0 ± 1.4								Li (#1)
	Sign. 3	•	•	•			16.7 ± 1.9								Li (#2)
	Sign. 4	•	•	•	•		16.3 ± 1.4								Va (#1)
	Sign. 5	•	•	•	•	•	16.0 ± 1.0								Li (#3, #5)
															Va (#3, #4)
															Te (#1, #2)
	Sign. 6		•	•	•	•	15.4 ± 0.8								Va (#5)
															Te (#3)
	Sign. 7			•	•	•	14.9 ± 0.5								Te (#5)
	Sign. 8				•	•	14.7 ± 0.9								Ma (#2, #3)
															Va (#2)
															Te (#4)
	Sign. 9					•	14.5 ± 0.8								Ma (#1, #4, #5)
*Porites rus*															
*Cladocopium*	Sign. 1		•				17.2 ± 0.6								Te (#4)
	Sign. 2		•	•			16.7 ± 0.3								Te (11)
	Sign. 3		•	•	•		16.4 ± 0.5								Li (#1, #3, #4, #5)
															Va (#1, #4, #5)
															Ma (#2, #4, #5)
															Te (#3, #5)
	Sign. 4			•	•		16.0 ± 0.4								Ma (#1, #3)
															Te (#2)
															Va (#2)
	Sign. 5				•		15.7 ± 0.6								Li (#2)
*Cladocopium* > *Symbiodinium*	Sign. 6			•	•		15.9 ± 0.4						•	5.8 ± 1.0	Va (#3)
*Acropora cytherea*															
*Durusdinium > Symbiodinium*	Sign. 1	•					19.7 ± 1.2	•						16.1 ± 1.4	Li (#4)
	Sign. 2	•					20.1 ± 1.0		•					14.0 ± 1.2	Va (#4)
	Sign. 3	•					19.9 ± 1.3				•			9.2 ± 1.4	Li (#2)
	Sign. 4	•	•				19.3 ± 0.9		•	•				12.4 ± 1.1	Va (#5), Te (#4)
	Sign. 5	•	•	•			18.8 ± 0.6		•					13.6 ± 0.6	Te (#6)
	Sign. 6	•	•	•			17.9 ± 1.6			•	•			11.3 ± 0.6	Va (#1)
	Sign. 7		•	•	•		17.4 ± 1.6		•	•				13.2 ± 2.1	Va (#2)
	Sign. 8			•	•		16.8 ± 1.2		•	•				12.6 ± 1.1	Te (#5)
	Sign. 9				•		15.2 ± 2.1		•	•				13.2 ± 1.3	Va (#3)
*Symbiodinium*	Sign. 10							•	•					14.5 ± 1.6	Te (#1)
	Sign. 11								•					13.7 ± 0.9	Li (#1, #5)
*Durusdinium*	Sign. 12	•					20.0 ± 0.4								Li (#3)
	Sign. 13	•	•	•			18.9 ± 2.3								Te (#3)
*Cladocopium*	Sign. 14	•					19.6 ± 1.5								Te (#2)

Reef sites are coded as follows: Linareva (Li), Teavaro (Te), Vaiare (Va) and Maharepa (Ma).

### Coral hosts harbour temporary and/or sporadic Symbiodiniaceae genera

The abundance and diversity of non-permanent Symbiodiniaceae genera (temporary and/or sporadic) was highly variable among coral species in the study period. The majority of *P. acuta* colonies harboured sporadic clades (18/20), in contrast with less than half of the colonies in *P. rus* (9/20) and *A. cytherea* (7/16) (chi-squared test: *χ*^2^ = 11.19 df = 2, *P* = 0.004). Despite the stability of the *Durusdinium*-pattern observed in *P. acuta*, this coral species was simultaneously flexible in its ability to associate with up to three additional sporadic genera (*Symbiodinium*, *Breviolum* and *Cladocopium* simultaneously in Pac1 at Li; or individually with *Symbiodinium* in Pac2 from sites Li and Va; *Breviolum* in Pac1 and Pca5 at site Ma; and *Cladocopium* in 17 colonies; Fig. 2). Similarly, *P. rus* displayed a stable *Cladocopium*-pattern with occasional sporadic *Symbiodinium* (n = 7; e.g. Pru2 at Li and Te, Pru4 at Ma) and/or *Durusdinium* (n = 3; i.e. Pru1 at Te, Pru5 at Va, Pru1 at Ma). *Acropora cytherea* had complex Symbiodiniaceae community patterns with occasional sporadic *Symbiodinium* (n = 2; Acy2 and Acy3 at Te), *Cladocopium* (n = 4; e.g. Acy4 at Li, Te and Va, Acy5 at Te) and/or *Durusdinium* (n = 1; Acy1 at Te). Nearly all of the sporadic Symbiodiniaceae genus detections appeared with S/H ratio quantities much lower than the ones detected in permanent genera, especially for *P. acuta* and *P. rus*.

In contrast to sporadic genera, temporary genera were only harbored by low numbers of colonies, and their frequency did not significantly differ among coral species: *P. acuta* (1/20; Pac2 at Li), *P. rus* (5/20; Pru3, Pru4 and Pru5 at Li and Pru1 and Pru4 at Va) and *A. cytherea* (3/16; Acy3 at Te, Acy2 and Acy5 at Va) (chi-squared test: *χ*^2^ = 3.08, df = 2, *P* = 0.214). Temporary clades had a low diversity in all coral species: only *Cladocopium* in *P. acuta* (Pac2 at Li), only *Symbiodinium* in *P. rus* (n = 5 colonies), and *Cladocopium* or *Symbiodinium* in *A. cytherea*, in three colonies (e.g. Acy3 at Te and Acy5 at Va) and one colony (Acy3 at Li), respectively.

### Dynamics of Symbiodiniaceae genera in response to environmental variation

**Figure 3:**
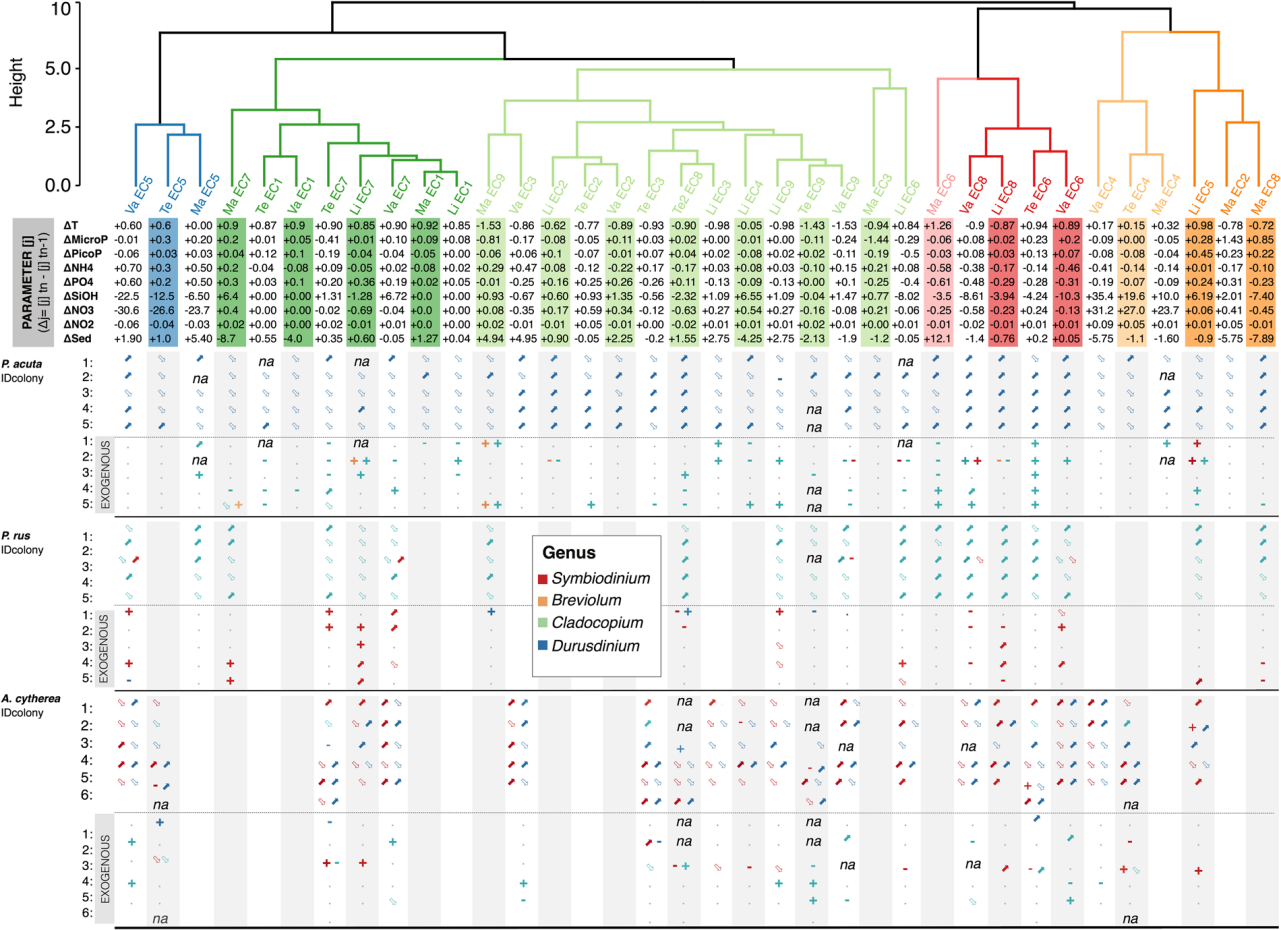
Results of the cluster analysis of environmental contexts from reef sites Linareva (Li), Teavaro (Te), Maharepa (Ma) and Vaiare Va) and the corresponding dynamics of Symbiodiniaceae genera associated to *Pocillopora acuta* (PAC), *Porites rus* (PRU) and *Acropora cytherea* (ACY) from February 2011 to August 2012, between two successive sampling times *tn+1* and *tn*. The Hierarchical Clustering Analysis was applied on the matrix with delta values of each environmental parameters using the Euclidean distance coefficient to compare ECs and Ward’s method of minimum variance to assemble clusters (i.e. package ‘pvclust’ in R 101). ECs are coded according to the chronological sampling periods as follows: EC1 (Feb11-Apr11), EC2 (Apr11-Jun11), EC3 (Jun11-Aug11), EC4 (Aug11-Oct11), EC5 (Oct11-Dec11), EC6 (Dec11-Feb12), EC7 (Feb12-Apr12), EC8 (Apr12-Jun12) and EC9 (Jun12-Aug12). The analysis is based on the quantitative variation of the different environmental parameters of sea surface temperature (T), Phytoplankton >2 μm (Micro) and <2 μm (PicoP), ammonium (NH_4_^+^), phosphate (PO_4_^3-^), silicate (SIOH), nitrite (NO_3_^-^), nitrate (NO_2_^-^) and sedimentation (Sed), described in^63^. The Symbiodiniaceae genus dynamics recorded into different tagged coral hosts dissociated with their label number (IDcol) from different species are described through their quantitative variation (increase: ‘↑’, decrease: ‘↓’, loss: ‘−’ or re-acquisition: ‘+’) of permanent clades and/or non-permanent clades (exogenous exchanges with temporary and sporadic clades).

The permanent Symbiodiniaceae genus-patterns of each coral species were similar among study sites, despite the environmental contrasts (Fig. 3). In particular, no significant correlation was detected between the S/H ratio of Symbiodiniaceae genera and any environmental parameter or sites.

There were no common patterns in Symbiodiniaceae genus dynamics (S/H ratio variation during a period = Δ_genus i, n_) among and within species when correlated to clusters of environmental contexts, except for EC8 (Figs 2 and 3). This environmental context cluster was characterized by a decrease in sediment rates, a decrease in sea surface temperature (T) and nutrient concentrations (i.e. ammonium [NH_4_^+^], phosphate [PO_4_^3−^], silicates [SiOH], nitrates [NO_3_^−^] and nitrites [NO_2_^−^]) and an increase in phytoplankton concentrations (micro- and pico- fractions). Indeed, between April and June 2012, there was a significant interaction between site and sampling time for the permanent genus *Durusdinium* in *P. acuta* and a significant effect of sampling time for *Cladocopium* in *P. rus* (Table S1). At that period, we observed a significant increase in *Durusdinium* in *P. acuta* at all four reef sites (Fig. 3). A pairwise analysis revealed that the variations of sea surface temperature, PO_4_^3−^, SiOH and NO_2_^−^ were significantly and negatively correlated with changes in the abundance of *Durusdinium* (Fig. S2a). EC8 was also significantly associated with changes in the abundance of *Cladocopium* in *P. rus*, which increased in nearly all tagged colonies (17/20) from undisturbed or disturbed sites, and the pairwise analysis showed that PO_4_^3−^ drove this negative correlation (Fig. S2b).

In contrast, the permanent genus *Symbiodinium* and/or *Durusdinium* S/H ratio in *A. cytherea* did not show any significant variations with respect to EC (Fig. S2c).

The overall distribution of non-permanent genera did not significantly differ from random patterns in the study period in any of the three coral species (*p* > 0.05 compared to Binomial and Poisson distributions, Supplementary Information SI 2). Variations of *Symbiodinium* abundance in *P. rus* were correlated to changes in SST, PO_4_^3−^ and NH_4_^+^ (Fig. S2b), however the dynamics of temporary or sporadic genera (appearance or disappearance; Fig. 2) did not show clear patterns in relation to any particular environmental change (within or between ECs, Fig. 3) for any of the species (Fig. S1a–c).

## Discussion

The choice of the qPCR assay employed in the present study was based on its ability to detect Symbiodiniaceae genera *in hospite* at the level of ≤200 28S copies, which approximates the number of 28S copies in a single algal cell^50^. The combination of this highly sensitive method with a sampling every two months of 56 tagged colonies of three coral host species enabled us to identify putative inferences of both newly acquired Symbiodiniaceae genera (switching) and shifts in the abundance of genera (shuffling) over a period that spans multiple seasons. The overall dataset provides a comprehensive picture of the diversity and quantitative variations of Symbiodiniaceae in various coral hosts over time and in different environments.

### Random acquisition and short-term maintenance of Symbiodiniaceae

The detection of *de novo* Symbiodiniaceae genera in all three coral species suggests that symbiont switching may be a common natural phenomenon in healthy adult scleractinians. Interestingly, while clade F was presents in the surrounding environment^50^, it was never detected in coral samples, indicative of an active control of symbiont uptake by the host. Previous studies suggested that Symbiodiniaceae switching occurs readily in juvenile stages^23^, while in adults it only happens when the health of the colony is compromised during infected experimental conditions^49,65^ or *in situ*^36^. While exogenous symbiont acquisition may be more common in healthy adult corals than previously thought, the rate at which it occurs varies substantially among species. Of the three coral species studied here, *P. acuta* showed a significantly higher affinity to integrate sporadic clades compared to *P. rus* and *A. cytherea*. This interspecific difference may reflect divergent strategies for coping with fluctuating environmental conditions. A higher flexibility in symbiont switching in *P. acuta* may be a biological mechanism adapted to compensate for the dominance of thermally tolerant but low efficiency genus *Durusdinium*^66–70^ in this coral’s microbiome. Supportive of the symbiont switching hypothesis our study also found that six out of twenty (i.e. 30%) *Cladocopium*-dominated *P. rus* colonies acquired a background population of *Symbiodinium* over several consecutive months at some study reef sites but not for all surveyed colonies and without any temporal patterns. These results contradict previous reports^71–73^ that describe an exclusive association of *P. rus* with a single ITS2 genotype (C15 of genus *Cladocopium*) and could suggest a potential ecological role of the cryptic *Symbiodinium* in this coral species. Thus, while it is impossible to rule out that some of the *de novo* genera are merely consumed Symbiodiniaceae that are not in symbiosis with the coral (see^47^), these can also be the first steps of ‘symbiont switching’ (Fig. 2). Although apparently not driven by external factors (within the study period), such random sporadic and/or temporary symbionts switching might strongly impact the fitness and behavior of coral colonies. This has been previously shown for *A. cytherea* in which the external acquisition of *Durusdinium* was correlated by the loss of a *Vibrio* *spp.*^36^. Furthermore, our results suggest that the mechanism of acquisition and the maintenance of non-permanent Symbiodiniaceae genera is largely labile (e.g. change in Pattern Recognition Receptors, or the lack of their maintenance^74,75^), colony dependent and extends the mechanism of symbiont acquisition/selection already observed in symbiont-free offspring^76^.

### Dynamics of permanent Symbiodiniaceae genera

Variations of the abundance of permanent Symbiodiniaceae genera in correlation with environmental changes suggests that the holobiont can rapidly modulate its autotrophic activity by increasing its Symbiodiniaceae densities to counterbalance conditions for limited heterotrophy. For example, one event of putative Symbiodiniaceae shuffling was observed between April and June 2012 in all tagged *P. acuta* (*Durusdinium*) colonies and at all four reef sites, as well as in *P. rus* (*Cladocopium*) colonies at the Linareva (undisturbed) and Vaiare (disturbed) sites. This period was characterized by a decrease in sea surface temperature and in nutrient concentrations (i.e. inorganic nitrogen, phosphate, silicates) and an increase in phytoplankton concentration (micro- and pico- fractions). Previous reports (reviewed in^77^) stated that increased inorganic nitrogen levels boost Symbiodiniaceae densities under high irradiance conditions, as they are normally nitrogen-limited, while increased inorganic phosphate does not have a major effect. Therefore, the negative correlation detected between inorganic phosphate and Symbiodiniaceae densities is unexpected and could be explained by the fact that our study was conducted *in situ*, or that other parameters not measured here drive symbiont dynamics. Alternatively, this result suggests novel mechanisms currently not fully understood.

Only one potential shuffling event was detected in this study that supports the ABH^39,61^. Indeed, there were no clear patterns in symbiont dynamics in response to environmental changes, i.e. neither for changes in one particular environmental parameter or the combination of multiple parameters. This is consistent with previous ecological observations showing that even if coral species are able to host a large diversity of symbionts, they will not necessarily activate rapid partnership changes as a response to acute environmental change^36,47,49,65,78^. Although changes in the abundance of non-permanent Symbiodiniacea genera were observed in all studied coral species, our results indicate that these acquisitions are random and their maintenance limited, supporting the idea that even under environmental changes, established symbioses are highly stable over time with only transient modifications^47^. This finding has also been highlighted in a recent reciprocal transplant experiment along a depth gradient^78^ in which corals that vertically transmit their symbionts reversed after several months to the original symbiotic communities even if these were well-suited to the transplantation depth.

### Long term stability of permanent clades: coral colonies with unique Symbiodiniaceae signatures

The stability and host specificity for particular Symbiodiniaceae genera has been well-established for most coral taxa in both tropical^36,44–46,78,79^ and high-latitudinal^47,80,81^ regions. In the present study, both coral species that vertically transmit their symbionts (i.e. from parent to offspring) displayed strict host specificity for a single permanent clade (*Durusdinium* in *P. acuta* and *Cladocopium* in *P. rus*), whereas the horizontally transmitting coral species *A. cytherea* exhibited multiple host clade-patterns (*Durusdinium*/*Symbiodinium*, *Symbiodinium*, *Durusdinium* or *Cladocopium*). These findings corroborate the hypothesis that the reproductive strategy of corals plays a key role in the establishment of host-symbiont associations, with more specific and stable patterns characteristic for vertically transmitting ‘symbiont specialist’ corals^45,82–85^, compared with the enhanced flexibility often encountered in horizontally transmitting or ‘symbiont generalist’ corals^80,83,85^.

Most notably, this study also reveals that the coral-Symbiodiniaceae specificity is a trait that is shaped at the colony scale. Permanent Symbiodiniaceae genera are regulated by the host in a non-random manner, within a specific density range, which we coined ‘Symbiodiniaceae signature’. This signature reflects high intraspecific variability in symbiont communities in all three-coral species, which enables distinguishing individual coral colonies by their microbiome. In non-stressful conditions of symbiosis the Symbiodiniaceae densities are regulated in the host at an equilibrium state to sustain both partners in a position of mutual benefit^86^. Therefore, based on the diversity of Symbiodiniaceae signatures observed in the present study, we suggest that the symbiosis equilibrium state might be host dependent. This host regulation can be synergistically affected by environmental filtering. For example, *P. acuta* and *A. cytherea* from the undisturbed reef of Linareva had higher *Durusdinium* quantities in their Symbiodiniaceae signatures. It is potentially one way for the hosts to compensate for the poor contribution of *Durusdinium* symbionts to the holobiont’s metabolism, as previously described in non-stressful conditions^53,68,69^. The control of Symbiodiniaceae community composition can be reached through intrinsic and extrinsic factors (reviewed in^87,88^), including direct expulsion^64,89^ or *in hospite* degradation of Symbiodiniaceae cells (e.g. necrosis, apoptosis;^87^). Regardless of the control mechanism involved, each individual coral colony harbors a specific suite and abundance of Symbiodiniaceae genera that likely reflect on their unique plasticity in endosymbiont regulation.

Our findings indicate that each holobiont is characterized by a host with a specific Symbiodiniaceae signature of permanent genera and a constitutive flexibility to associate with additional temporary or sporadic genera. In the context of the current model of Symbiodiniaceae-coral winnowing^14,90–94^, this new theory of Symbiodiniaceae signatures suggests the existence of regulatory mechanisms within the host that enable them to maintain and regulate their permanent Symbiodiniaceae genera homeostasis within a particular range. When identical genera are present but in different quantity ranges, this signature can be considered as distinct amounts of Symbiodiniaceae ‘genomes’ modulating the hologenome (*sensu*^95^), which could account for differences in the host’s behavior^92,96^. Additionally, the appearance of sporadic and/or temporary non-permanent genera in the host may contribute to the diversity of the coral hologenome (see^95^). This diversity may contribute to explaining intraspecific differences in the ecological success of distinct holobionts (Hologenome Dependent Susceptibility) and may constitute an important factor underpinning their resistance or resilience to environmental stressors.

As ocean warming progresses, more research on the ecological relevance of particular coral hologenomes, including the Symbiodiniacea signature within and among coral species (e.g. physiological or gene expression responses to multiple stressors), will be critically important to better predict the fate of coral reefs and to assist in the elaboration of effective conservation plans.

## Supplementary information

Supplementary data file
