# Supplementary material for: Unique quantitative Symbiodiniaceae signature of coral colonies revealed through spatio-temporal survey in Moorea
Source: Sci Rep. 2019 May 28;9:7921. doi: 10.1038/s41598-019-44017-5 (PMC6538640; doi:10.1038/s41598-019-44017-5)
Supplement: Supplementary file 1 — Supplementary data file [file 41598_2019_44017_MOESM1_ESM.pdf]

Unique quantitative Symbiodiniaceae signature of coral colonies revealed through spatio-temporal survey in Moorea

Héloïse Rouzé[1], Gaël Lecellier[2,3], Xavier Pochon[4,5], Gergely Torda[6] and Véronique Berteaux-Lecellier[1,2]

[1] USR3278 CRIOBE, Labex CORAIL, BP 1013 Papetoai, 98729 Moorea, French Polynesia

[2] UMR250/9220 ENTROPIE, Labex CORAIL, 101, promenade Roger-Laroque, BP A5 98848 Noumea cedex New-Caledonia.

[3] Université de Paris-Saclay, UVSQ, 55 Avenue de Paris, 78035 Versailles Cedex, France.

[4] Coastal and Freshwater Group, Cawthron Institute, 98 Halifax Street East, 7010 Nelson, New Zealand.

[5] Institute of Marine Science, University of Auckland, Warkworth 0941, New Zealand.

[6] Australian Research Council Centre of Excellence for Coral Reef Studies, James Cook University, Townsville, Australia.

Supplementary Information 1:

Supplementary figures

Figures S1-S2

Table S1-S2

Supplementary Information 2:

Supplementary figures

Figures SI 2

Figure S1 Temporal series of seawater temperatures recorded at the four reefs of Linareva (Li), Teavaro (Te), Vaiare (Va), and Maharepa (Ma) surveyed from February 2011 to August 2012

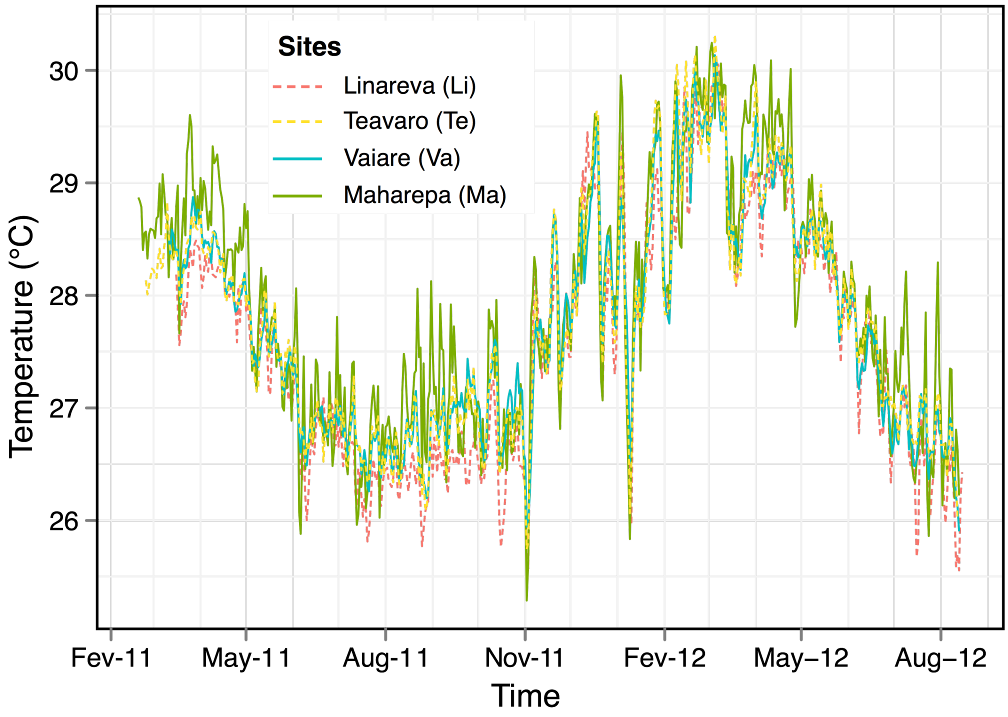

a. Pocillopora acuta

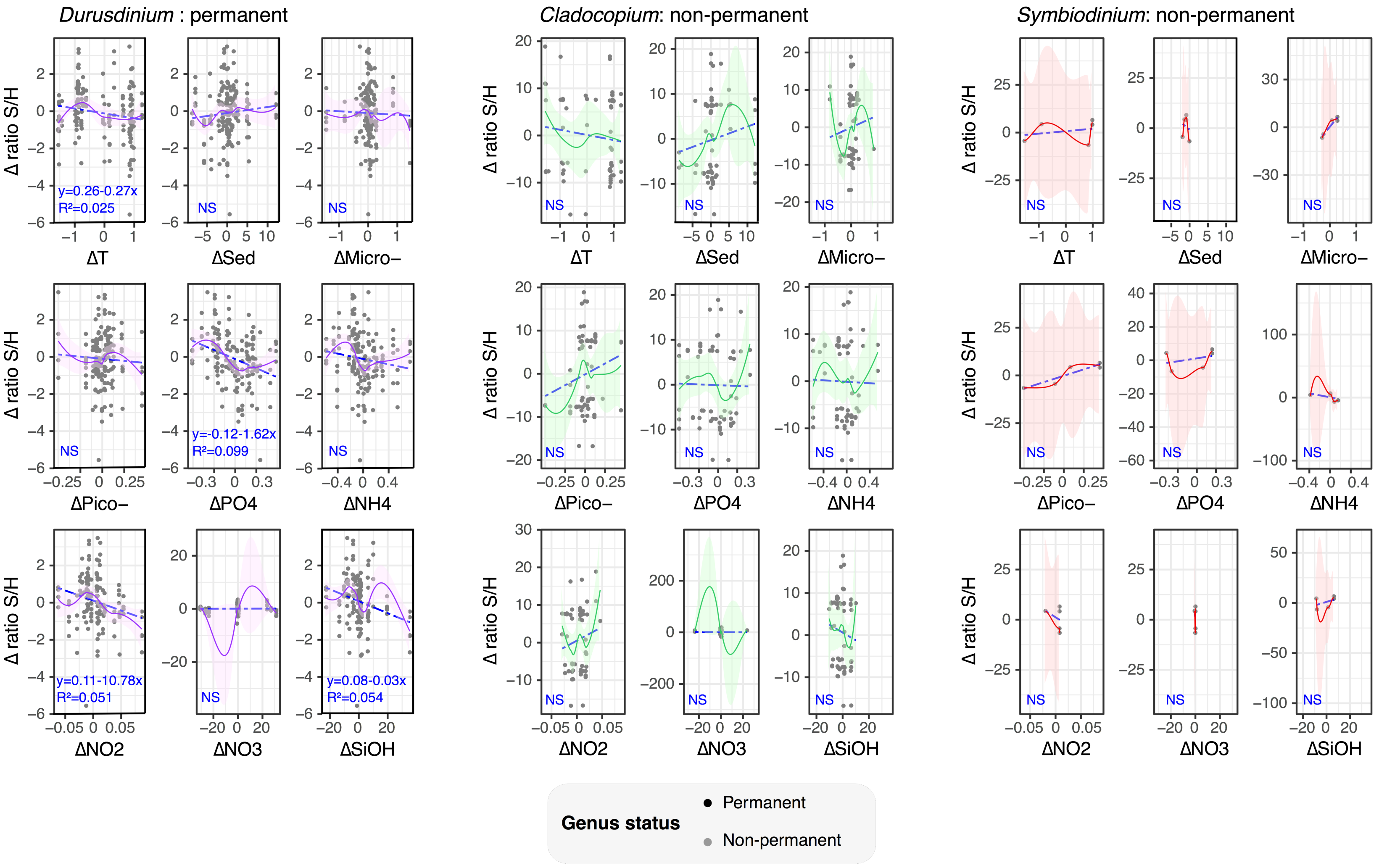

b. Porites rus

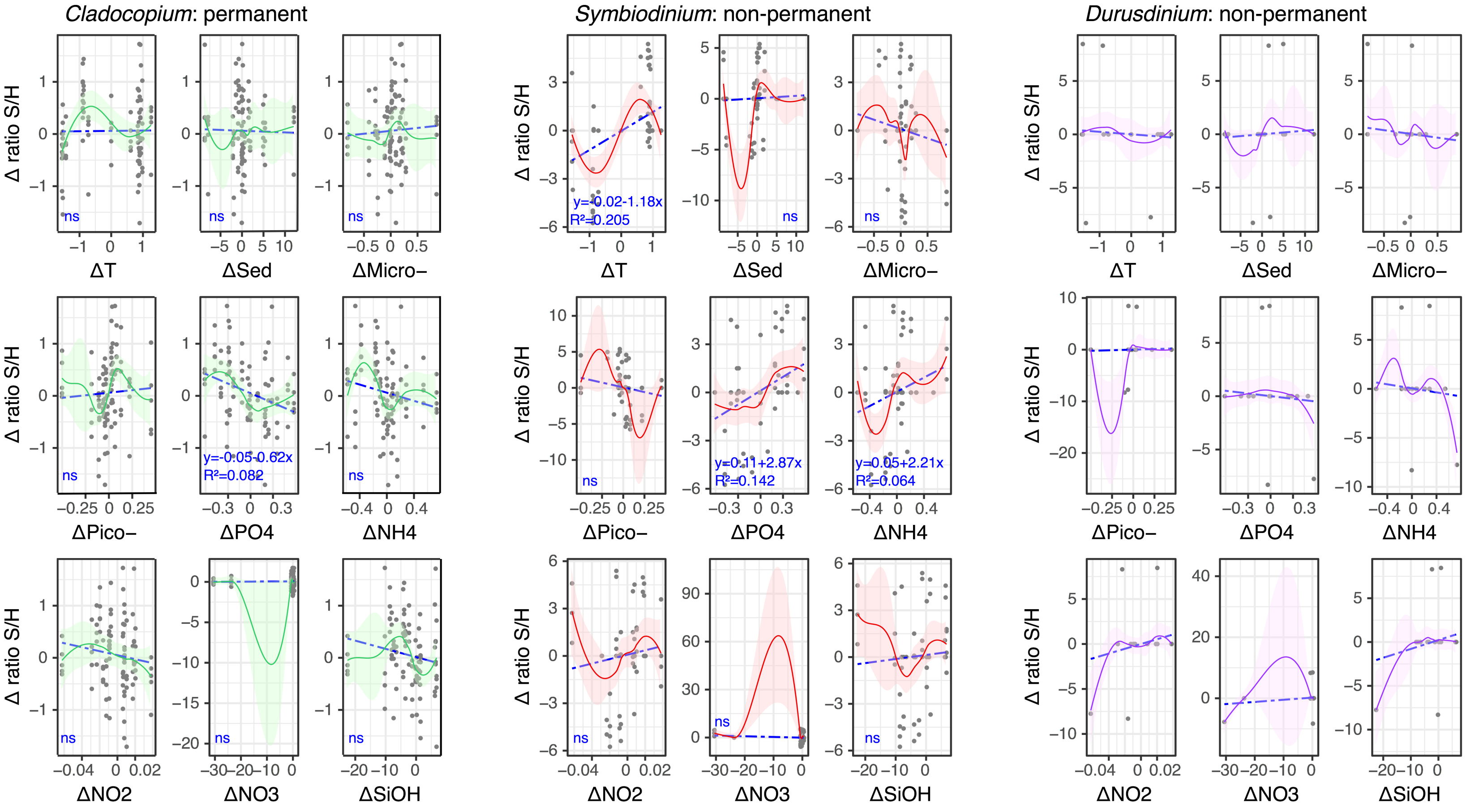

c. Acropora cytherea

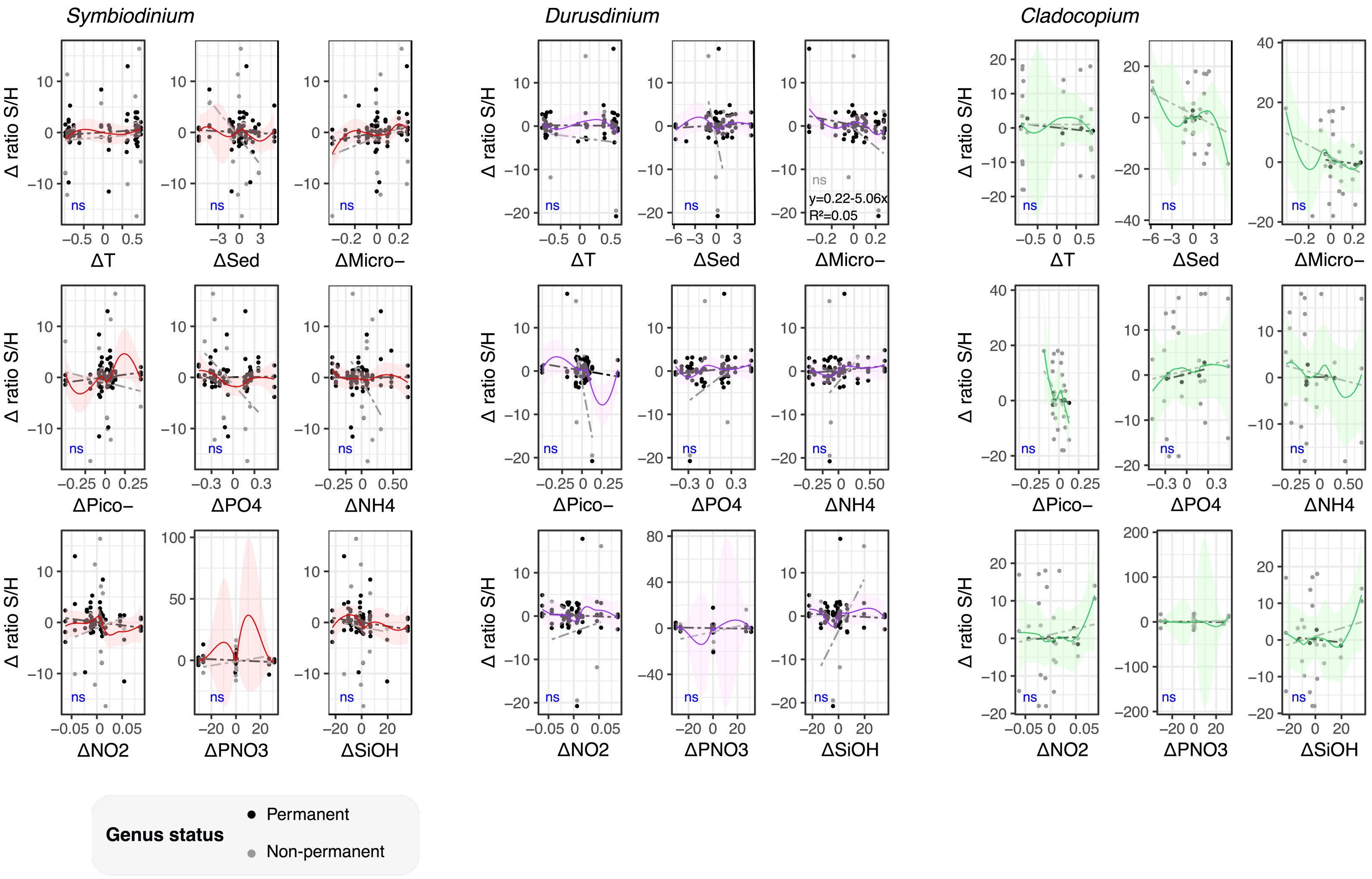

Figure S2 Linear regression models describing the variation between two successive sampling times t_n and t_{n-1} of *Symbiodinium, Cladocopium* and/or *Durusdinium* quantities in 28S copies per 18S polyp (H) (genus i: $\Delta_{genus\ i,n} = [S_i/H_{tn+1}] – [S_i/H_{tn}]$) and at particular environmental parameters values (parameter j: $\Delta_j= [j]t_{n+1} - [j]t_n$) in *Pocillopora acuta* (a.), *Porites rus* (b.) and *Acropora cytherea* (c.). The analysis is based on the quantitative variation of the different environmental parameters of sea surface temperature (ΔT), sedimentation (ΔSed), Phytoplankton >2 µm (ΔMicro) and <2 µm (ΔPico), phosphate (ΔPO4), ammonium (ΔNH4), nitrite (ΔNO3), nitrate (ΔNO2) and silicate (ΔSIOH) described in (Rouzé *et al.*, 2015).

Table S1 Results of two-way ANOVA showing the effects and interactions of space (SITE) and time (Period tn+1/tn) on the dynamics of permanent Symbiodiniaceae genera (genus i: Δgenus i,n between two successive sampling times tn+1 and tn) of Pocillopora acuta, Porites rus and Acropora cytherea. Reef sites are coded as follows: Linareva (Li), Teavaro (Te), Vaiare (Va) and Maharepa (Ma).

Species	Factor	Dominant Genus (code)	Genus-pattern(s)	df	F	P
P. acuta	Site	*Durusdinium* (D)	D	3	0.359	0.783
	Period (t_n-t_{n-1})			8	12.1	<0.001 ***
	Site x Period			24	2.615	<0.001 ***
P. rus	Site	*Cladocopium* (C)	C and CA	3	0.329	0.8
	Period (t_n-t_{n-1})			4	6.032	<0.001 ***
	Site x Period			11	1.795	0.07
A. cytherea	Site	*Symbiodinium* (A)	A and AD	2	0.035	0.97
	Period (t_n-t_{n-1})			6	0.652	0.69
	Site x Period			12	0.486	0.92
	Site	*Durusdinium* (D)	D and AD	2	0.061	0.94
	Period (t_n-t_{n-1})			6	0.088	0.99
	Site x Period			12	0.673	0.78

*** : p-value <0.001

Table S2 Mean values ± SD (standard deviation) of environmental parameters monitored during the spatio-temporal survey of the four fringing reef stations around Moorea. ND: means not detected because parameter was not assessed. From Rouzé et al. 2015

Location	Parameter	Feb (T0)	Apr (T1)	Jun (T2)	Aug (T3)	Oct (T4)	Dec (T5)	Feb (T6)	Apr (T7)	Jun (T8)	Aug (T9)
		Wet season 2011		Dry season 2011			Wet season 2012			Dry season 2012	
	Micro- (µg/L)	0.06 ± 0.00	0.15 ± 0.00	0.06 ± 0.01	0.07 ± 0.01	0.06 ± 0.01	0.35 ± 0.05	0.06 ± 0.01	0.06 ± 0.00	0.07 ± 0.00	0.06 ± 0.01
	Pico- (µg/L)	0.09 ± 0.01	0.09 ± 0.00	0.18 ± 0.02	0.21 ±0.01	0.13 ± 0.01	0.56 ± 0.05	0.08 ± 0.00	0.04 ± 0.00	0.06 ± 0.00	0.05 ± 0.01
	Sediment (mg/d/cm^2)	1.99 ± 0.73	2.62 ± 1.47	3.50 ± 0.77	6.02 ± 1.22	2.38 ± 1.06	1.07 ± 0.31	1.20 ±0.30	1.81 ± 0.38	0.91 ± 0.52	0.91 ± 0.37
	NH_4^+ (µM)	0.11 ± 0.01	0.13 ± 0.01	0.05 ± 0.00	0.09 ± 0.00	0.17 ± 0.01	0.17 ± 0.04	0.26 ± 0.08	0.21 ± 0.02	0.04 ± 0.01	0.11 ± 0.01
Linareva	PO_4^{2-} (µM)	0.32 ± 0.04	0.37 ± 0.01	0.54 ± 0.09	0.41 ± 0.06	0.32 ± 0.07	0.56 ± 0.2	0.12 ± 0.09	0.53 ± 0.13	0.23 ± 0.01	0.23 ± 0.01
undisturbed	NO_3^{2-} (µM)	ND	0.19 ± 0.04	0.37 ± 0.01	0.80 ±0.53	1.20 ± 0.04	1.25 ± 0.03	1.23 ± 0.13	0.51 ± 0.08	0.29 ± 0.03	0.75 ± 0.04
	NO_2^- (µM)	ND	0.03 ± 0.01	0.01 ± 0.00	0.02 ± 0.01	0.03 ± 0.00	0.04 ± 0.01	0.05 ± 0.00	0.04 ± 0.01	0.03 ± 0.01	0.07 ± 0.00
	SiOH (µM)	ND	1.89 ± 0.06	2.47 ± 0.03	3.97 ± 1.50	10.16 ± 0.30	15.42 ± 1.74	8.28 ± 0.27	7.27 ± 1.76	3.07 ± 0.03	3.29 ± 0.06
	Precipitation (mm)	347.8 ± 10.36	169.7 ± 6.12	194.4 ± 6.30	55.0 ± 3.26	216.6 ± 11.70	349.0 ± 17.71	586.8 ± 21.36	313.0 ± 10.82	298.1 ± 15.15	153.6 ± 8.89
	T° (°C)	27.24 ± 0.37	28.17 ± 0.41	27.45 ± 0.62	26.53 ± 0.40	26.52 ± 0.39	27.40 ± 0.10	28.25 ± 0.85	29.18 ± 0.61	28.28 ± 0.61	26.70 ± 0.57
	Micro- (µg/L)	0.10 ± 0.02	0.12 ± 0.00	0.07 ± 0.01	0.11 ± 0.02	0.11 ± 0.03	0.38 ± 0.04	0.61 ± 0.03	0.20 ± 0.01	0.22 ± 0.02	0.11 ± 0.00
	Pico- (µg/L)	0.09 ± 0.03	0.21 ± 0.01	0.13 ± 0.01	0.21 ± 0.01	0.12 ± 0.02	0.16 ± 0.01	0.29 ± 0.02	0.10 ± 0.00	0.145 ± 0.00	0.04 ± 0.00
	Sediment (mg/d/cm^2)	2.08 ± 0.49	4.12 ± 2.45	3.76 ± 2.59	3.76 ± 3.39	1.96 ± 2.49	2.13 ± 1.20	3.67 ± 3.57	3.23 ± 2.11	4.20 ± 0.42	2.30 ± 0.40
	NH_4^+ (µM)	0.22 ± 0.02	0.18 ± 0.01	0.06 ± 0.00	0.23 ± 0.02	0.09 ± 0.02	0.39 ± 0.01	0.25 ± 0.02	0.31 ± 0.09	0.18 ± 0.01	0.24 ± 0.11
Teavaro	PO_4^{2-} (µM)	0.32 ± 0.03	0.35 ± 0.05	0.60 ± 0.03	0.36 ± 0.06	0.30 ± 0.07	0.45 ± 0.02	0.24 ± 0.15	0.40 ± 0.06	0.25 ± 0.01	0.22 ± 0.02
Undisturbed	NO_3^{2-} (µM)	ND	0.30 ± 0.01	0.56 ± 0.10	0.67 ± 0.36	28.21 ± 1.79	0.90 ± 0.01	1.16 ± 0.05	1.12 ± 0.62	0.50 ± 0.05	0.69 ± 0.14
	NO_2^- (µM)	ND	0.01 ± 0.00	0.05 ± 0.01	0.05 ± 0.01	0.11 ± 0.01	0.06 ± 0.00	0.06 ± 0.00	0.08 ± 0.04	0.06 ± 0.01	0.04 ± 0.00
	SiOH (µM)	ND	2.85 ± 1.37	3.06 ± 0.10	2.64 ± 0.32	23.58 ± 3.22	8.49 ± 0.09	4.45 ± 0.44	6.52 ± 1.60	3.28 ± 0.00	3.12 ± 0.03
	Precipitation (mm)	186.3 ± 6.75	53.2 ± 2.63	226.0 ± 11.62	45.4 ± 2.83	98.4 ± 4.8	480.9 ± 19.7	593.7 ± 21.4	482.6 ± 23.29	253.5 ± 11.38	129.3 ± 7.34
	T° (°C)	27.51 ± 0.54	28.39 ± 0.31	27.56 ± 0.59	26.71 ± 0.44	26.86 ± 0.52	27.48 ± 0.88	28.44 ± 1.02	29.27 ± 0.70	28.39 ± 0.66	26.96 ± 0.45
	Micro- (µg/L)	0.20 ± 0.03	0.25 ± 0.05	0.37 ± 0.06	0.19 ± 0.03	0.092 ± 0.01	0.07 ± 0.01	0.27 ± 0.02	0.32 ± 0.09	0.45 ± 0.07	0.21 ± 0.02
	Pico- (µg/L)	0.23 ± 0.03	0.34 ± 0.01	0.33 ± 0.02	0.39 ± 0.03	0.32 ± 0.01	0.26 ± 0.01	0.32 ± 0.01	0.26 ± 0.05	0.36 ±0.01	0.22 ± 0.05
	Sediment (mg/d/cm^2)	12.83 ± 3.65	9.70 ± 1.79	13.17 ± 3.21	16.78 ± 3.81	10.00 ± 6.76	13.51 ± 8.79	13.01 ± 8.38	12.03 ± 7.71	9.70 ± 4.53	9.43 ± 4.42
	NH_4^+ (µM)	0.39 ± 0.02	0.32 ± 0.05	0.09 ± 0.01	0.57 ± 0.10	0.16 ± 0.02	0.66 ± 0.15	0.43 ± 0.09	0.45 ± 0.03	0.07 ± 0.02	0.22 ± 0.03
Vaiare	PO_4^{2-} (µM)	0.39 ±0.06	0.52 ± 0.11	0.75 ± 0.09	0.55 ± 0.12	0.33 ± 0.07	0.86 ± 0.23	0.58 ± 0.13	0.76 ± 0.03	0.29 ± 0.03	0.39 ± 0.02
Disturbed	NO_3^{2-} (µM)	ND	0.45 ± 0.09	0.76 ± 0.05	0.41 ± 0.06	31.37 ± 1.00	1.17 ± 0.34	0.96 ± 0.05	0.91 ± 0.05	0.32 ± 0.05	0.39 ± 0.04
	NO_2^- (µM)	ND	0.05 ± 0.00	0.05 ± 0.02	0.03 ± 0.01	0.11 ± 0.00	0.05 ± 0.01	0.06 ± 0.02	0.07 ± 0.00	0.05 ± 0.00	0.05 ± 0.00
	SiOH (µM)	ND	2.03 ± 0.05	3.32 ± 0.10	2.75 ± 0.13	37.93 ± 0.70	15.76 ± 4.21	5.35 ± 0.38	11.97 ± 2.56	3.37 ± 0.03	4.82 ± 0.06
	Precipitation (mm)	186.3 ± 6.75	53.2 ± 2.63	226.0 ± 11.62	45.4 ± 2.83	98.4 ± 4.8	480.9 ± 19.7	593.7 ± 21.35	482.6 ± 23.29	253.5 ± 11.38	129.3 ± 7.34
	T° (°C)	27.7 ± 0.31	28.47 ± 0.38	27.54 ± 0.52	26.70 ± 0.27	26.91 ± 0.44	27.51 ± 0.77	28.43 ± 0.79	29.24 ± 0.55	28.34 ± 0.60	26.87 ± 0.41
	Micro- (µg/L)	0.13 ± 0.02	0.22 ± 0.02	1.46 ± 0.79	0.22 ± 0.01	0.17 ± 0.02	0.31 ± 0.04	0.26 ± 0.01	0.44 ± 0.02	1.24 ± 0.24	0.47 ± 0.06
	Pico- (µg/L)	0.10 ± 0.01	0.047 ± 0.02	0.31 ± 0.18	0.10 ±0.01	0.05 ± 0.00	0.08 ±0.00	0.05 ± 0.01	0.08 ±0.01	0.29 ±0.09	0.25 ± 0.00
	Sediment (mg/d/cm^2)	ND	9.00 ± 1.58	3.28 ± 0.31	1.90 ± 0.35	3.53 ± 0.97	12.38 ± 8.94	20.18 ± 4.31	12.55 ± 3.59	4.20 ± 1.35	9.00 ± 1.44
	NH_4^+ (µM)	0.33 ± 0.08	0.25 ± 0.03	0.09 ± 0.00	0.29 ± 0.02	0.16 ± 0.02	0.59 ± 0.08	0.12 ± 0.01	0.32 ± 0.01	0.23 ± 0.02	0.51 ± 0.02
Maharepa	PO_4^{2-} (µM)	0.32 ± 0.03	0.36 ± 0.02	0.67 ± 0.04	0.47 ± 0.12	0.35 ± 0.08	0.80 ± 0.17	0.21 ± 0.02	0.53 ± 0.13	0.27 ± 0.02	0.26 ± 0.03
Disturbed	NO_3^{2-} (µM)	ND	0.30 ± 0.01	0.71 ± 0.07	1.04 ± 0.24	24.63 ± 0.67	0.90 ± 0.18	0.78 ± 0.18	1.08 ± 0.02	0.64 ± 0.00	0.71 ± 0.05
	NO_2^- (µM)	ND	0.02 ± 0.01	0.07 ± 0.00	0.06 ± 0.01	0.09 ± 0.01	0.06 ± 0.01	0.051 ± 0.01	0.06 ± 0.00	0.06 ± 0.00	0.08 ± 0.00
	SiOH (µM)	ND	1.42 ± 0.09	3.39 ± 0.08	4.16 ± 1.21	14.78 ± 1.21	7.53 ± 1.41	4.09 ± 0.31	13.09 ± 5.34	3.19 ± 0.05	4.20 ± 0.28
	Precipitation (mm)	314.0 ± 12.6	126.3 ± 4.5	270.4 ± 9.4	67.8 ± 2.76	151.9 ± 6.58	666.8 ± 30.06	702.8 ± 25.95	520.1 ± 15.57	452.3 ± 19.2	117.3 ± 5.05
	T° (°C)	28.03 ± 1.05	28.77 ± 1.44	27.70 ± 1.18	26.79 ± 0.95	27.13 ± 1.15	27.50 ± 1.33	28.51 ± 1.46	29.51 ± 1.21	28.46 ± 1.32	27.05 ± 1.13

Supplementary information 2

The observed global distribution of *Cladocopium* in *P. acuta* did not significantly differ from either the binomial (P =0.2) or Poisson (Lambda=2) distributions (Fig.SI a). Additionally, the distribution of *Cladocopium* was not significantly affected by site (Pearson's Chi-squared: χ=13, df = NA, P = 0.1709) or by season (Pearson's Chi-squared: χ^2=8.4375, df = 5, P = 0.1337)

However, the observed global distribution of *Symbiodinium* in *P. rus* significantly differed from both the binomial and Poisson (P <0.05 for both) distributions (Fig.SI b). The distribution was significantly affected by the site (Pearson's Chi-squared: χ^2=16.133, df = 3, P = 0.001065).

a. *P. acuta:* non-permanent *Cladocopium*

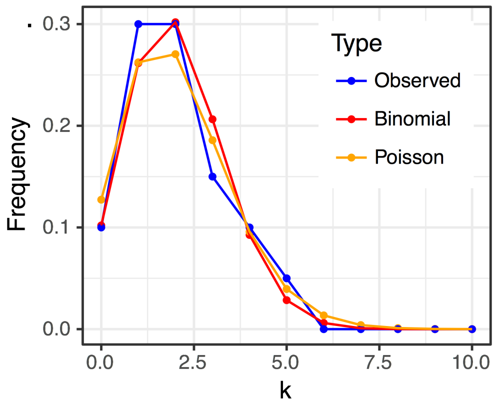

b. *P. rus:* non-permanent *Symbiodinium*

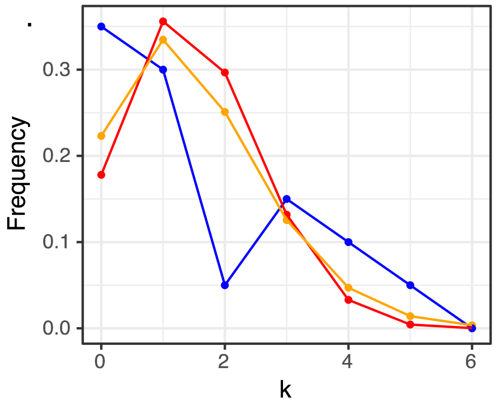

Fig.SI2 Global distribution of non-permanent Symbiodiniaceae genus *Cladocopium* in *P. acuta* and *Symbiodinium* in *P. rus* compared to Binomial and Poisson distributions.
